# Management of hazardous fly-ash energy waste in the adsorptive removal of diclofenac by the use of synthetic zeolitic materials

**DOI:** 10.1007/s11356-022-24619-9

**Published:** 2022-12-21

**Authors:** Magdalena Medykowska, Małgorzata Wiśniewska, Katarzyna Szewczuk-Karpisz, Rafał Panek

**Affiliations:** 1 Department of Radiochemistry and Environmental Chemistry, Institute of Chemical Sciences, Faculty of Chemistry, Maria Curie-Sklodowska University, Maria Curie-Sklodowska Sq. 3, 20-031 Lublin, Poland; 2 Institute of Agrophysics, Polish Academy of Sciences, Doświadczalna 4, 20-290 Lublin, Poland; 3 Department of Building Materials Engineering and Geoengineering, Faculty of Civil Engineering and Architecture, Lublin University of Technology, Nadbystrzycka Street 40, 20-618 Lublin, Poland

**Keywords:** Waste management, Water purification, Diclofenac adsorption/desorption, Zeolitic materials, Adsorbent-adsorbate interactions, Polyacrylic acid addition, Solid aggregation

## Abstract

Zeolite-carbon composites (Na-P1(C), Na-X(C)) and pure zeolites (Na-P1, Na-X) were synthesized from hazardous high-carbon fly ash waste (HC FA) via hydrothermal reaction with sodium hydroxide (NaOH). These solids were applied in the removal of diclofenac (DCF) from aqueous solution, with and without poly(acrylic acid) (PAA). The experiments included adsorption–desorption measurements, as well as electrokinetic and stability analyses. The obtained results showed that HC FA and Na-P1(C) had the greatest adsorption capacity towards DCF, i.e., 26.51 and 21.19 mg/g, respectively. PAA caused considerable decrease in the DCF adsorption due to the competition of both adsorbates of anionic character for active sites. For example, the adsorbed amount of DCF on Na-P1 without PAA was 14.11 mg/g, whereas the one measured with PAA was 5.08 mg/g. Most of prepared solids were effectively regenerated by the use of NaOH. Desorption degree reached even 73.65% in the single systems (with one adsorbate) and 97.24% in the mixed ones (with two adsorbates). Zeolitic materials formed suspensions of rather low stability, which underwent further deterioration in the organic molecules presence. All the results obtained in this study indicated that HC FA can be successfully managed in the removal of organic substances.

## Introduction

Hazardous waste can be generated in various areas including agriculture, industry, and household. Many toxic substances affecting the quality of the environment and human life, both liquids and solids, end up in wastewater, which must then be properly treated. Due to the different origin, wastewater can be divided into several categories, e.g., domestic, industrial, wastewater from institutions, stormwater, and that generated inside a wastewater treatment plant (Correia et al. 1994; Akarsubasi et al. 2005). All those types may contain microorganisms (pathogenic bacteria, viruses or worm eggs), metals (including heavy metals such as lead, mercury, zinc, nickel, chromium), inorganic compounds (acids, bases), radioactive isotopes, and various organic substances (pesticides, fertilizers, drugs, dyes) (Chen et al. 2020; Huang et al. 2010; Fu and Wang 2011).

Pharmaceuticals are characterized by a very diverse chemical structure and properties. Thus, they are difficult to remove and considered as highly hazardous components of wastewater. In addition to active substances, drugs can also contain various types of dyes and fillers. Since only a few of them are metabolized by organisms, they often end up in the wastewater and the environment unchanged. In various ecosystems, released antibiotics and other pharmaceuticals contribute to the formation of drug-resistant pathogenic strains. Over-the-counter non-steroidal anti-inflammatory drugs such as diclofenac (DCF), which are used in large quantities and then excreted by humans, are also a serious problem (Kümmerer 2001; Yamashita et al. 2006; Zuccato et al. 2005). It has been estimated that the annual global consumption of DCF is 940 tons (Zhang et al. 2008).

One of the ways to dispose of drugs from wastewater is the activated sludge process. It is a process of biological purification through the passage of microorganisms and decomposition of matter in specially supercharged reactors. The use of biologically active sand filters, adsorption on carbonaceous materials and hybrid techniques, such as ultrasound/H_2_O_2_, ultrasound/ozone or ozone/H_2_O_2_ processes, are also popular (Carvalho et al. 2013; Petrie et al. 2013; Gogate and Pandit 2004; Camargo-Perea et al. 2020). The methods for effective and inexpensive removal of anti-inflammatory drugs are constantly being developed. Many researchers focused on diclofenac. Li et al. (2020) used novel red mud (RM-PPy) to remove DCF. Its adsorption capacity in the single adsorbate system was 195 mg/g, whereas in the mixed one it was 115.7 mg/g. Viotti et al. (2019) used biosorbents prepared from Moringa oleifera pods and activated carbon from Babassu coconut. The maximum adsorption capacity towards DCF of the first one was approximately 60.805 mg/g, whilst that of the second one was 71.150 mg/g. In addition to anti-inflammatory drugs, there are also literature reports on antibiotic and dye removal. Mohammadi et al. (2022) investigated the removal capacity of the antibiotic amoxicillin by zero-valent iron-oxide nanoparticles in the presence of hydrogen peroxide. Through optimization, they removed 99.7% of this antibiotic from aqueous solutions. Berizi et al. (2016) examined magnetite nanoparticles and magnetite nanoparticles modified with sodium alginate for the removal of Acid Red 18. The maximum capacity of the unmodified material was 16.259 mg/g, in turn that of the modified adsorbent was 73.464 mg/g. Azari et al. (2017) removed 2,4-dichlorophenol from aqueous systems using Magnetic Graphene Oxide Particles (MGO NPs). They were able to remove 100% of this compound (84.74 mg/g). Yousefzadeh et al. (2017) removed diethylphthalate using AnFFBR and UAnFFFBR. The removal rates for these materials were 91.11 and 88.72%, respectively. Badi et al. (2019) performed experiments using the UVC/SPS/Fe^2+^ system and noted high levels of dimethylphthalate removal from tap water, surface runoff as well as treated and raw wastewater (95.7, 88.5, 80.5 and 56.4%, respectively).

Zeolites are materials that can also bind large amounts of impurities on their surface. They are hydrated aluminosilicates of metals from I and II group, formed in hydrothermal processes of rock transformation in the environment (e.g., linoptilolite, mordenite and garronite) or synthesize in laboratory (e.g., zeolite A, P, X and Y). Natural zeolites are characterized by better resistivity and thermal stability in the different environments than synthetic ones (Khaleque et al. 2020). On the other hand, synthetic zeolites have larger pore volume and thus greater adsorption capacity relative to toxic metal ions, radioactive wastes and other contaminants. Synthetic zeolites are generally prepared from different natural or man-made raw materials using various techniques such as hydrothermal method, alkali-fusion method, sol–gel method, and alkali-leaching method (Sugano et al. 2005; Wajima et al. 2008; Shoppert et al. 2017). Among all these techniques, due to its simplicity and low cost, hydrothermal method is most often applied. In this method, water is used as a solvent, and base is used as a mineralizer. The reaction is performed a sealed vessel usually made of polypropylene and teflon-lined (PTFE) steel autoclave (Sangeetha and Baskar 2016).

In this study, zeolite-carbon composites and pure zeolites were obtained from hazardous fly ash precursor by hydrothermal method. Also, prepared materials and their precursor were applied in the process of diclofenac removal from aqueous solution. To reflect the complexity of natural samples, the studies were carried out not only in single systems containing one adsorbate, but also in mixed ones containing both DCF and poly(acrylic acid) (PAA). PAA is a common constituent of wastewater and surface water, as it is widely used as a soil conditioner as well as a thickener in pharmaceutical or cosmetic industries. During the experiments, the maximum adsorption capacity, adsorption kinetics, as well as electrokinetic and stability properties of the systems were investigated. The presented issues are innovative and described in the literature for the first time. So far, zeolite-carbon composites have not been described as adsorbents of diclofenac in the presence of a macromolecular compound. Literature reports on the removal of two organic substances at the same time are scarce. The obtained results indicate the possibility of managing the troublesome waste produced during the combustion of hard coal, i.e., fly ash.

## Experimental

### Materials

In the experiments, 5 solids were applied. There were:

1. High-carbon fly ash (HC FA),
2. Pure zeolites Na-X and Na-P1,
3. Zeolite-carbon composites Na-X(C) and Na-P1(C).

High-carbon fly ash, produced in conventional hard coal combustion, was taken from the Janikowo thermal power plant. It was used to prepare zeolite-carbon composites and pure zeolites via hydrothermal reaction, which involves sodium hydroxide (NaOH, CAS 1310–73-2). The obtained solids were fully characterized in terms of elemental composition, morphology, mineral composition, surface properties, aggregate size, and porous structure in our previous papers (Panek et al. 2021b, c). The textural parameters of zeolitic materials (ASAP 2020 apparatus, Micromeritics Instrument Corporation, Norcross, GA, USA) are presented in Table 1.

**Table 1:** Textural parameters of zeolitic adsorbents (S_BET_ the BET specific surface area, S_m_ the micropore area, V_m_ the micropore volume, V_t_ the pore volume, D mean pore diameter) (Panek et al. 2021b, c)

Sample	S_BET_ [m^2^/g]	S_m_ [m^2^/g]	V_t_ [cm^3^/g]	V_m_ [cm^3^/g]	D [nm]
HC FA	46.2	10.7	0.06	0.006	5.7
Na-P1(C)	69.8	16.9	0.12	0.007	6.7
Na-P1	26.7	4.3	0.05	0.002	6.9
Na-X(C)	271.9	188.8	0.17	0.07	2.6
Na-X	727.9	694.1	0.31	0.27	1.7

Diclofenac sodium salt (DCF, CAS 15307–79-6, Sigma-Aldrich) was used in the study as an adsorbate. In turn, pol(acrylic acid) (PAA, CAS 9003–01-4, Sigma-Aldrich) was applied as a substance affecting the DCF adsorption. Both organic substances have anionic character resulting from the occurrence of carboxyl groups in their molecules. These moieties show weak acidic properties and undergo dissociation with the increase in solution pH value. The physicochemical characteristics of DCF and PAA are presented in Table 2 (Gebhardt and Furstenau 1983; Chibowski et al. 2003; Jodeh et al. 2016; Lach and Szymonik 2020).

**Table 2:** Physicochemical characteristics of diclofenac and poly(acrylic acid) (M_w_ the molecular weight, pKa the negative base 10 log of the acid dissociation constant Ka)

Sample	Molecular formula	Structural formula	M_w_ [g/mol]	pK_a_
DCF	C_14_H_10_Cl_2_NNaO_2_		318.1	4.15
PAA	(CH_2_-CHCO_2_H)_n_		240 000	4.5

Sodium chloride (NaCl, CAS 7647–14-5, Sigma Aldrich) with the concentration 0.001 mol/dm^3^ was applied in all experiments as a supporting electrolyte. Hydrochloric acid (HCl, CAS 7647–01-0) or NaOH with the concentrations of 0.1 mol/dm^3^ were used to carry out the desorption process, as well as for pH adjustment.

### Methods

#### Production of zeolitic materials

Zeolite-carbon composites were prepared from HC FA in the hydrothermal reaction with NaOH performed according to the following parameters. For Na-P1(C), 20 kg of HC FA was mixing with 90 dm^3^ of 3 M NaOH at 90 °C for 24 h. For Na-X(C), 25 kg of HC FA was mixing with 90 dm^3^ of 3 M NaOH at 80 °C for 48 h (Bandura et al. 2021). As a result of these reactions, a solid product in the form of zeolitic material and a liquid waste in the form of post-reaction solution were obtained. The second one contained great amounts of NaOH and dissolved aluminosilicate glaze consisting mainly of silicon (Si) and aluminum (Al). This solution was the substrate for the preparation of pure zeolites according to the procedure developed by Panek et al. (2021a). In this case, the hydrothermal reaction with different parameters was exploited. For Na-P1, 40 cm^3^ of the waste solution was mixed with 10 cm^3^ of NaOH and 80 g of aluminum foil at 100 °C for 48 h. For NaX, 50 cm^3^ of the waste solution was mixed with 50 cm^3^ of NaOH and 450 g of aluminum foil at 70 °C for 24 h. The applied NaOH concentration was 3 mol/dm^3^. The obtained pure zeolites had a monophasic structure with no ash residues.

#### Electrokinetic study

The potentiometric titrations were performed in the systems containing 0.4 g of HC FA, 0.03 g of Na-X, 0.4 g of Na-P1, 0.075 g Na-X(C) or 0.2 g of Na-P1(C) in 50 cm^3^ of the supporting electrolyte solution. The concentration of DCF and PAA was 50 ppm. The surface charge density (σ_0_) and point of zero charge (pzc) of the examined suspensions were determined by automatically controlled potentiometric titration set (thermostated Teflon vessel, glass and calomel electrodes (Beckman Instruments), pH-meter PHM 240 (Radiometer), laboratory stirrer, thermostat RE 204 (Lauda), automatic microburette Dosimat 765 (Metrohm)) using the computer program “titr_v3”. The difference in the volume of the titrant (NaOH with the concentration 0.1 mol/dm^3^) added to the suspension and the supporting electrolyte solution providing a specific pH value was determined and applied to the σ_0_ calculations (Janusz 1994).

The electrophoretic mobility (U_e_) measurements were performed in the systems containing 0.005 g of the solid and 100 cm^3^ of the supporting electrolyte solution. The concentration of DCF and PAA was 50 ppm. These experiments enabled the calculation of the zeta potential (ζ) and the determination of isoelectric point (iep) of the examined suspensions. The zetameter Nano ZS (Malvern Instruments, Cambridge, UK) equipped with immersion dip cell was used. After the 3-min sonication, the prepared suspension was divided into eight parts and a specific pH value (in the range 3–10) was adjusted in each of them. Then, the electrophoretic mobilities were measured and using the Henry equation, the zeta potential value was calculated (Oshima 1994).

#### Adsorption/desorption study

Diclofenac adsorbed amounts on pure zeolites, zeolite-carbon composites, and fly ash were determined based on the difference in adsorbate concentration in the solution before and after the adsorption. At the beginning, the series of samples (10 cm^3^) were prepared by addition of 0.01 g of the solid to the solution containing supporting electrolyte and diclofenac with the concentration of 10–90 ppm. Then, the pH value of the systems was adjusted to 5, and the adsorption process was conducted for 24 h (to ensure the equilibrium in the examined samples), under continuous shaking conditions. After the adsorption completion, the suspensions were filtered through paper and syringe filters, and then analyzed towards the diclofenac concentration using high-pressure liquid chromatography (Ultimate 3000, Dionex) equipped with DAD detector.

The kinetics of diclofenac adsorption on zeolites, zeolite-carbon, and fly ash was also investigated. At first, the suspensions (10 cm^3^) were prepared by addition of 0.01 g solid to the solution containing supporting electrolyte and diclofenac with concentration 50 ppm. After pH adjustment to the value of 5, the adsorption was conducted in the time period 0.5–15 h. After these times, the concentration of diclofenac was analyzed in the same way as during adsorption isotherm determination.

To estimate the poly(acrylic acid) impact on diclofenac adsorption on the examined solids surfaces, the adsorption was also performed in the mixed systems. For this purpose, the suspension contained 0.01 g of the solid, supporting electrolyte, 50 ppm of diclofenac, and 50 ppm of PAA (added simultaneously) were prepared. The adsorption was conducted at pH 5, for 24 h, under continuous shaking conditions. After solid separation, the concentration of diclofenac in the solutions was measured in the same way as during adsorption isotherm determination.

The solids with adsorbed DCF were taken for desorption study. At first, the solids were flooded with 10 cm^3^ of HCl or NaOH solutions with concentration 0.1 mol/dm^3^. Then, the prepared systems were shaken for 1 h. After the solid separation, the concentration of DCF was measured in the obtained clear solutions.

#### Stability study

The stability of zeolites, zeolite-carbon composites, and fly ash suspensions, with and without diclofenac and poly(acrylic acid), was estimated based on turbidimetric measurements (turidimeter 2100 AN, Hach). In the first step, the samples (20 cm^3^) were prepared using 0.05 g of the solid and supporting electrolyte. Then, after 3-min sonication, diclofenac and/or poly(acrylic acid) were added and the pH of the systems was adjusted to 5. The turbidity of the suspensions was measured after 0, 2, 5, 10, 20, 30 and 60 min.

The particle/aggregate sizes of zeolite and zeolite-carbon composites were measured using CPS analyzer (CPS 24 000, CPS Instruments). The samples were prepared in the same way as for turbidimetric measurements. The size of particles/aggregates was determined in the 0.01–5 μm range. The sucrose solutions with the concentrations of 8% and 24% were used in the gradient formation. In turn, the speed of disc was equal to 6000 rpm.

## Results and discussion

### The structure of electrical double layer formed on the zeolitic materials surface with and without diclofenac or/and poly(acrylic acid)

The structure of electrical double layer (EDL) formed at the solid/liquid interface in the presence of organic substances of big molecules (as DCF) or long chains (as PAA) differs significantly from the EDL structure formed on bare solid particles. In such a case, the composition of surface layer of EDL (characterized by the solid surface charge density and corresponding point of zero charge) can be completely different from the composition of slipping plane area (characterized by zeta potential and corresponding isoelectric point). It is typical behavior observed for many colloidal suspensions containing big molecules of drugs, pesticides or dyes, and polymeric macromolecules. The value of the zeta potential informs about the charge accumulated within the slipping plane, which is also a part of EDL located at some distance away from the surface layer.

**Fig. 1:**
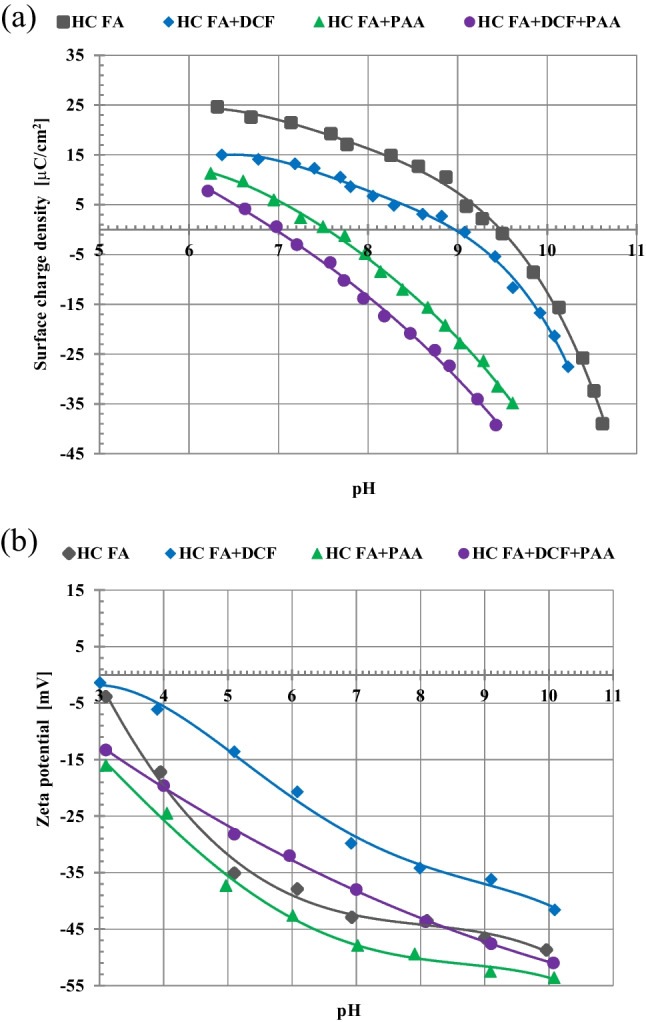
Surface charge density (**a**) and zeta potential (**b**) of HC FA particles as a function of solution pH with and without DCF or/and PAA (C_DCF_ = 50 ppm, C_PAA_ = 50 ppm)

The sign and magnitude of the charge occurring on the solid surface decide about its adsorption properties towards the organic molecules, such as DCF and PAA. On the other hand, the magnitude of charges present in the area of slipping plane around the solid particles (the specific value of zeta potential) affects the stability of suspension. The surface charge density and zeta potential measured for HC FA particles as a function of solution pH without and with DCF or/and PAA are presented in Fig. 1. For the composites and the pure zeolites, the similar tendencies were obtained. Table 3 summarizes the points of zero charge (pzc) of zeolitic adsorbents with and without adsorbates.

**Table 3:** Points of zero charge (pzc) of zeolitic adsorbents with diclofenac (DCF) or/and poly(acrylic acid) (PAA)

Sample	pzc without adsorbates	pzc with DCF	pzc with PAA	pzc with DCF and PAA
HC FA	9.5	9.0	7.5	7.0
Na-P1(C)	8.8	7.6	7.3	6.8
Na-P1	9.4	8.6	7.3	6.9
Na-X(C)	8.6	7.8	7.3	6.8
Na-X	9.0	8.5	8.0	7.5

In the absence of organic substances, the values of pzc of the examined solids changes from 8.6 for Na-X(C) to 9.5 for HC FA. At pH < pzc, the solid surface is positively charged. Thus, at pH 5, at which adsorption–desorption studies are performed, there are favorable electrostatic interactions between positive solid surface and negatively charged DCF and PAA molecules. Under these conditions, the dissociation of carboxyl groups present in DCF and PAA is significant and exceeds 50% at pH 5 (for DCF, the pK_a_ is 4.15, and for PAA, 4.5, Table 2) and, as a result, their adsorption causes the noticeable decrease in the solid surface charge density in the whole examined pH range (Fig. 1a). The points of zero charge of all soilds are also different, that is, shifted towards the lower pH values (Table 3). The greatest reduction in the surface charge density is obtained in the simultaneous presence of both adsorbates (DCF + PAA), whereas the smallest decrease in this parameter occurs in the systems with DCF.

Generally, the adsorption of small negatively charged ions and molecules increases surface charge density of the examined solid due to the creation of the additional number of positively charged surface groups (Skwarek et al. 2014; Wiśniewska et al. 2018; Fijałkowska et al. 2020a). In the case of long polymeric chains and large drug molecules of anionic character, the decrease in the solid surface charge is usually observed. The negatively charged functional groups of adsorbed organic molecules, whose are located in by-surface layer of the solution and are more numerous than those directly interacting with the solid surface, are responsible for this phenomenon (Wiśniewska et al. 2015, 2016; Fijałkowska et al. 2020b).

Zeta potential of zeolitic materials is higher in the presence of DCF for all examined systems. However, in the presence of both adsorbates, i.e., DCF + PAA, its values are lower than those noted for the suspension without adsorbates. For almost all tested systems (except Na-X and Na-P1(C) without adsorbates), zeta potential assumes only negative values. This makes it impossible to determine isoelectric points (iep) of the solids. In the case of Na-X and Na-P1(C), the iep points are 4.6 and 4.0, respectively.

**Fig. 2:**
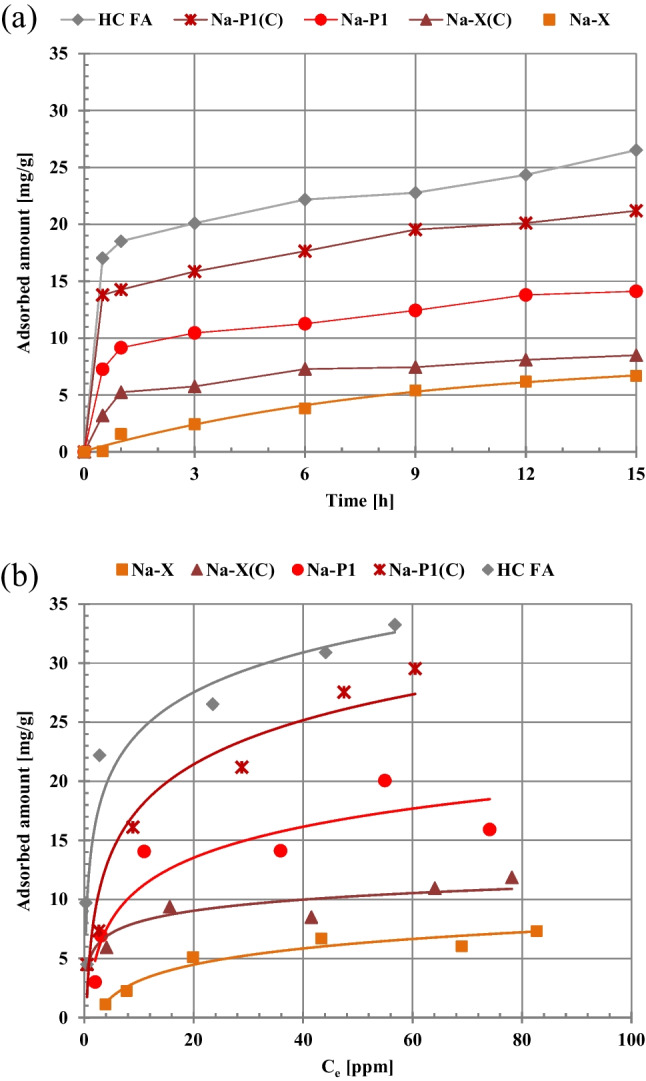
Adsorption kinetics (**a**) and (**b**) isotherms of DCF molecules on the fly ash precursor, zeolite-carbon composites and pure zeolites

The changes in the zeta potential of the solid particles in the presence of large organic molecules (polymers, drugs) are the result of three main effects (M’Pandou and Siffert 1987). The first one is displacement of the counter-ions located in the Stern layer due to adsorption of organic molecules causing desorption of supporting electrolyte ions from the solid surface and their removing from the surface layer towards the diffusion part of electrical double layer. The second one is the presence of negatively charged functional groups of the adsorbed molecules in the area of slipping plane, and the third effect is shift of the slipping plane by the adsorbed molecules or polymeric chains from the solid surface towards the bulk solution. The obtained electrokinetic results (Fig. 2b) indicate that the first phenomenon is dominant in the case of DCF. The adsorption of this substance causes effective displacement of supporting electrolyte Na^+^ cations from the stiff surface layer to the movable diffusion part of edl. For the suspension containing PAA, the shift of slipping plane and presence of negatively charged carboxyl groups of the polymer (each segment of flexible polymeric chain contain such a group) in this area are dominant.

### The adsorption–desorption mechanisms of diclofenac on the zeolitic materials surface – poly(acrylic acid) influence

The results of adsorption kinetics of diclofenac molecules are presented in Fig. 2a. The equilibrium state for all zeolitic materials was reached after relatively long time — about 6 h. This is probably the result of complicated structure and complex composition of the examined adsorbents.

The adsorption isotherms of DCF on the surfaces of fly ash precursor, zeolite-carbon composites, and pure zeolites are shown in Fig. 2b. As can be seen, the greatest adsorption of diclofenac is obtained on the surface of hazardous HC FA waste material (it reaches level about 33 mg/g for initial DCF concentration 90 ppm). Moreover, the zeolite-carbon composites have better adsorption abilities towards organic molecules compared to the corresponding pure zeolites. The adsorption capacities of the examined solids decrease in the following order: HC FA > Na-P1(C) > Na-P1 > Na-X(C) > Na-X. Despite the fact that the Na-X zeolite possesses the most developed specific surface area, its micropores of mean size 1.7 nm (Table 1) are available for adsorbing DCF molecules (with size is 0.97 × 0.98 nm and area – 0.52 nm^2^ (Sotelo et al. 2014; Bhadra et al. 2017)) to a limited extent and the adsorption reaches a level about 7 mg/g. Besides electrostatic attraction, the hydrogen bonds between -COO^−^ and -NH groups of diclofenac and hydroxyl groups of zeolitic solids can be formed (Wiśniewska et al. 2019a). In the case of Na-X(C) composite, the maximum adsorbed amount is higher and equal to about 11 mg/g due to the presence of additional hydrophobic interactions. The π-π electron donor–acceptor interactions can also occur between aromatic rings of drug molecules and carbon structures on the composite surface (Correa-Navarro et al. 2020; Szewczuk-Karpisz et al. 2021). These interactions seems to be more pronounced for the DCF molecules than those of hydrophilic character (i.e., hydrogen bonds). The considerable increase in the mean pore diameter in the case of Na-P1 zeolite, Na-P1(C) composite and HC FA precursor (6.9, 6.7 and 5.7 nm, respectively) results in noticeable greater adsorption of diclofenac due to the more effective penetration of the solid pores by its molecules. Nevertheless, in the case of HC FA precursor, there are probably the strongest π-π electron donor–acceptor interactions and the DCF adsorbed amounts are the highest. This makes it possible to realistically use of toxic waste from the energy sector to effective removal of drug molecules from the aqueous phase. Table 4 presents adsorbed amounts of DCF on the surface of other adsorbents as well as data obtained in this study.

**Table 4:** Comparison of the diclofenac adsorbed amounts on the surfaces of various solids

Adsorbent		Adsorption capacity	
The single systems			
GO-decorated CoFe_2_O_4_ CoFe_2_O_4_^a^		32.4 mg/g 18.4 mg/g	
Purified multi-walled carbon nanotubes (MWCNTs)^b^		19.9 mg/g	
Commercial gelatin/CNT’s beads RCTLW gelatin/CNT’s beads^c^		26.97 mg/g 20.57 mg/g	
Activated carbon from cocoa pod husk^d^		5.53 mg/g	
Alginate/Carbon films^e^		29.9 mg/g	
Cyclamen persicum tubers based activated carbon^f^		22.22 mg/g	
Activated carbon derived from orange peels K_2_CO_3_-activated carbon from orange peels^g^		6.44 mg/g 5.60 mg/g	
HC FA precursor Na-P1(C) Na-P1 Na-X(C) Na-X^h^		33.25 mg/g 29.53 mg/g 15.91 mg/g 11.86 mg/g 7.31 mg/g	
The mixed systems			
High surface area nanographene (HSANG)^i^	DCF with ibuprofen, ketoprofen, naproxen		19.3 mg/g
HC FA precursor			18.91 mg/g
Na-P1(C)	DCF with PAA		5.32 mg/g
Na-P1			5.08 mg/g
Na-X(C)			7.19 mg/g
Na-X^h^			5.86 mg/g

^a^ Van Tran et al. 2020, ^b^ Hu et al. 2017, ^c^ Rigueto et al. 2021, ^d^ De Luna et al. 2017, ^e^ Shamsudin et al. 2022, ^f^ Jodeh et al. 2016, ^g^ Tomul et al. 2019, ^h^ this study, ^i^Al-Khateeb et al. 2017

**Fig. 3:**
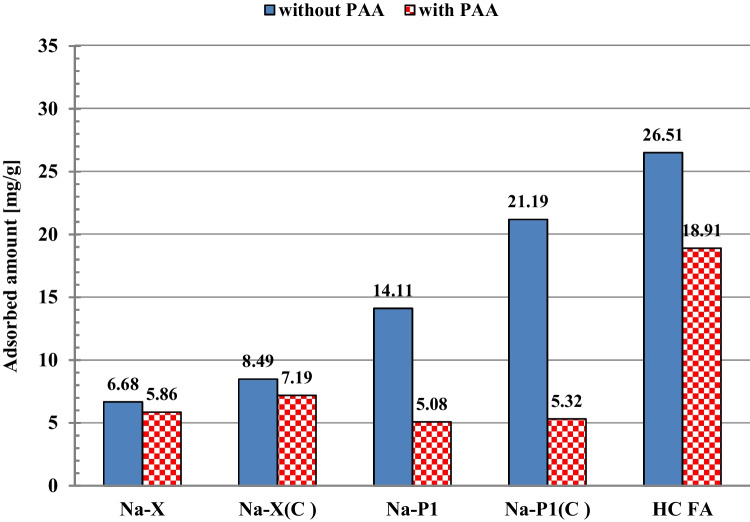
Adsorbed amounts of DCF on the ash precursor, zeolite-carbon composites and pure zeolites with and without PAA (C_0DCF_ = 50 ppm, pH 5, t_adsorption_ = 24 h)

The influence of poly(acrylic acid) on the adsorption of diclofenac on the surfaces of fly ash precursor, zeolite-carbon composites and pure zeolites is presented in Fig. 3. Its analysis leads to the conclusion that in all cases the PAA presence causes decrease in the DCF adsorbed amount. The greatest reduction in DCF adsorption due to the polymer presence is observed for the Na-P1(C) and Na-P1 systems. This phenomenon is certainly the result of competition of drug and polymer molecules, both of anionic character, for active sites present on the solid surface. At pH 5, polymeric chains are dissociated to a large degree and thus their conformations are more extended (Wiśniewska et al. 2013). For this reason, their adsorption on the positively charged solid surface causes effective blockade of its active sites making them inaccessible for diclofenac molecules.

**Fig. 4:**
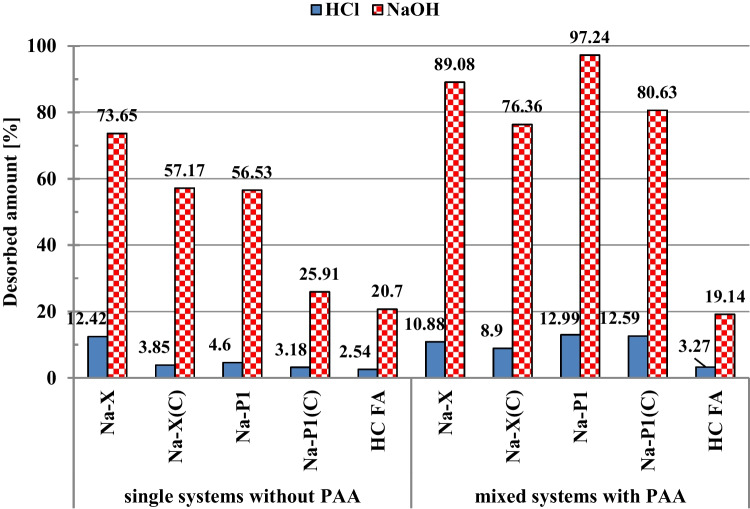
Desorbed amounts of DCF on the fly ash precursor, zeolite-carbon composites and pure zeolites with and without PAA (C_NaOH, HCl_ = 0.1 mol/dm.^3^, t_desorption_ = 1 h)

The regeneration possibilities of applied zeolitic adsorbents are presented in Fig. 4. Among used desorbing agents, the NaOH is considerably more efficient in comparison to HCl. This is probably due to the greater affinity of the sodium base to the acidic carboxyl groups of diclofenac (Wiśniewska and Nowicki 2020). The observed desorption is higher from mixed systems of adsorbates —for pure zeolites and their composites with carbon it changes in the range 76.36–97.24%. In such cases, the presence of PAA weakens even more the affinity of DCF for the surface of the solid. The smallest desorption percentages were obtained for the suspensions containing HC FA precursor, both in the single and mixed systems of adsorbates. This confirms quite strong binding of drug molecules on the surface of the starting material, which translates into its greatest adsorption abilities in relation to diclofenac.

### The stability of zeolitic materials suspensions in the presence of diclofenac or/and poly(acrylic acid)

**Fig. 5:**
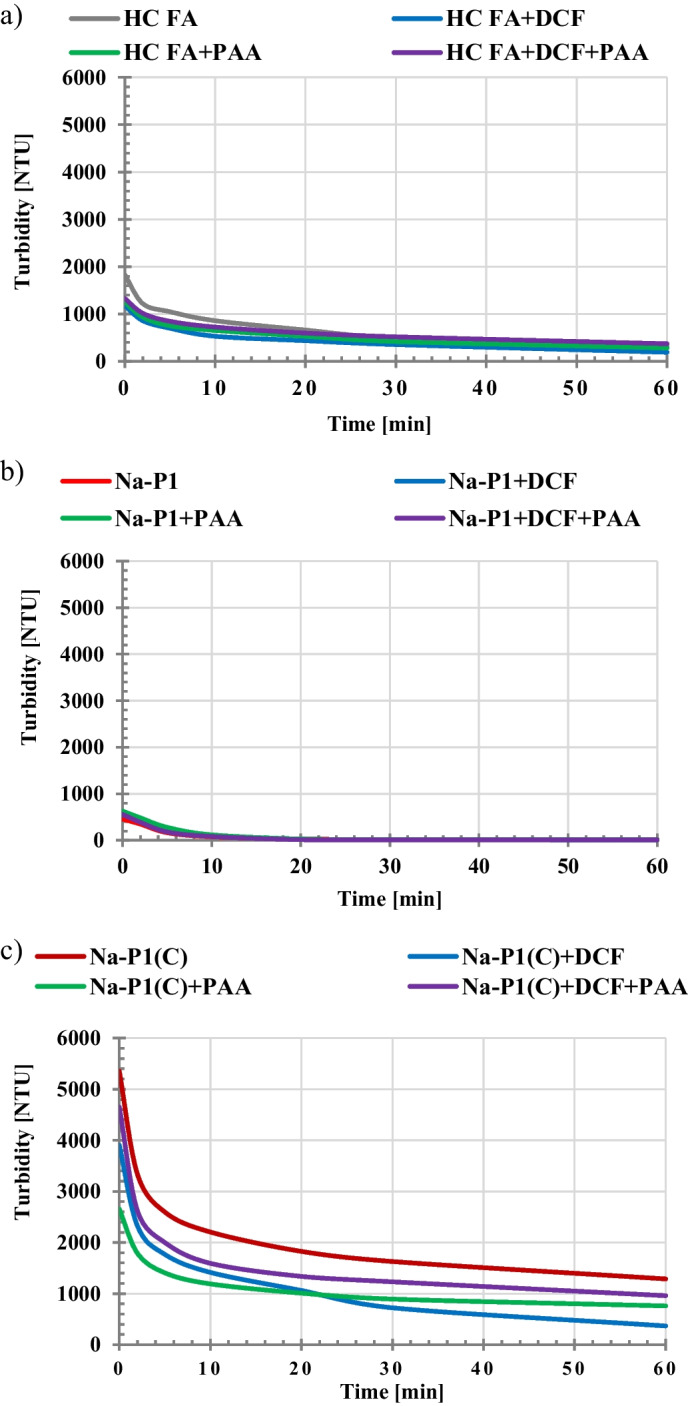
Turbidity of the suspensions of HC FA precursor (**a**), Na-P1(C) composite (**b**), Na-P1 zeolite (**c**) as a function of time with and without DCF or/and PAA (C_DCF_ = 50 ppm, C_PAA_ = 50 ppm)

**Fig. 6:**
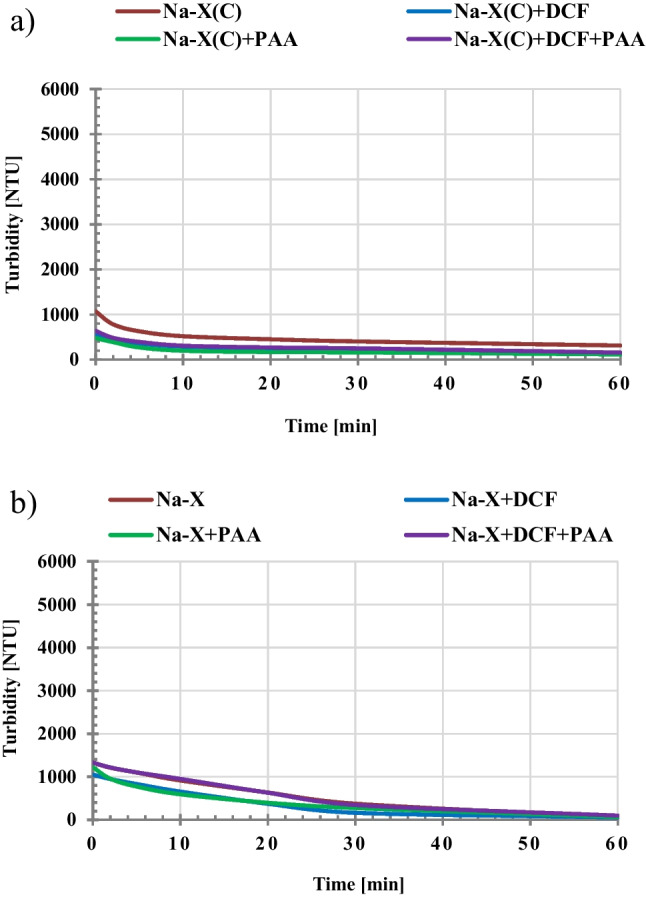
Turbidity of the suspensions of Na-X(C) composite (**d**) and Na-X zeolite (**e**) as a function of time with and without DCF or/and PAA (C_DCF_ = 50 ppm, C_PAA_ = 50 ppm)

**Fig. 7:**
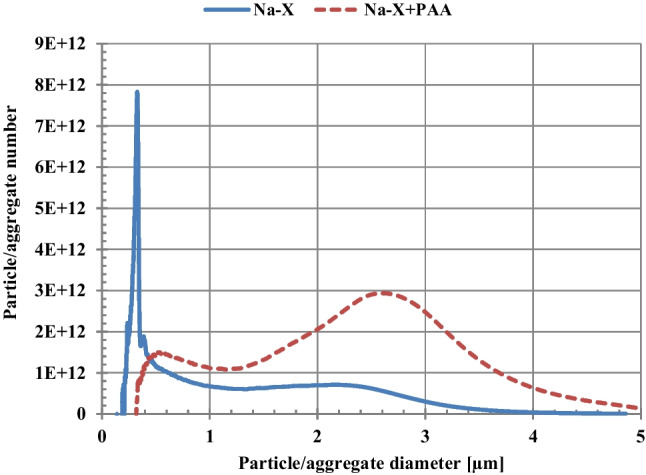
Size of formed aggregates/flocs of Na-X particles with and without PAA (CPAA = 50 ppm)

An important issue in the practical use of a given adsorbent, in addition to its adsorptive capacity and ability to regenerate, is also the easiness of its separation from the liquid phase. For this reason, the stability conditions in the examined suspensions without and with adsorbates, in their single and mixed systems, were determined. The obtained results of turbidity measurements are presented in Fig. 5. The most stable system among all tested ones turned out to be Na-P1(C) suspension (Fig. 5b), which was characterized by the highest values of turbidity (especially in the initial time period). In the case of this suspension, after addition of adsorbates, its stability undergoes deterioration. Other examined systems are unstable, and the presence of organic substances molecules results generally in further decrease in their stability. Such behavior is highly desired in effective separation of solid particles with adsorbed impurities from the liquid phase and makes this process much easier (Szewczuk-Karpisz et al. 2015). The decrease in the suspension stability in the presence of negatively charged diclofenac molecules and poly(acrylic acid) chains can be a result of two effects: (a) neutralization of positively charged solid particles by adsorbed DCF and PAA, as well as (b) formation of polymeric bridges between solid particles causing bridging flocculation. The latter mechanism is characteristic for long polymer chains which, by adsorbing on the surface of two or more colloidal particles, bind them together into large-sized flocs (Wiśniewska et al 2019b; Szewczuk-Karpisz et al. 2020). This is confirmed by the sizes of aggregates determined for the Na-X suspension without and with PAA (Fig. 6). The analysis of obtained results indicates that initial size of Na-X aggregates equal to 0.32 μm increases to the value 2.64 μm for Na-X/PAA flocs (Fig. 7).

## Conclusions

Fly ash-based zeolitic materials were examined as potential adsorbents of diclofenac in the poly(acrylic acid) presence. The obtained results showed that the greatest adsorption of diclofenac was on the surface of hazardous HC FA waste material. For initial DCF concentration 50 ppm, its adsorbed amount was 26.51 mg/g. The adsorption capacities of the examined solids decrease in the following order: HC FA > Na-P1(C) (21.19 mg/g) > Na-P1 (14.11 mg/g) > Na-X(C) (8.49 mg/g) > Na-X (6.69 mg/g). The greater affinity of drug molecules to the carbon-containing composites in comparison to the pure zeolites is caused by the occurrence of strong π-π electron donor–acceptor interactions between aromatic rings of DCF and carbon structures of the composites. Moreover, the adsorbed amounts of DCF increase with the increase of the solid mean pore size, which facilitates penetration of organic molecules to their interior. Poly(acrylic acid) causes decrease in the DCF adsorption due to the competition of both organic adsorbates endowed with negative charges. In the PAA presence, the DCF adsorbed amount was 18.91 mg/g for HC FA, 7.19 mg/g for Na-X(C), 5.86 mg/g for Na-X, 5.32 mg/g for Na-P1(C), and 5.08 mg/g for Na-P1. The obtained electrokinetic data proves that in the examined systems existed electrostatic attraction between adsorbates and adsorbents; nevertheless, the hydrophobic forces appearing for suspensions containing HC FA and carbon-zeolite composites were essential in the adsorption process. The examined materials can be regenerated using NaOH (desorption degree was even 97.24% for Na-P1 in the mixed system) and easily separated from the liquid phase due to their relatively low stability, especially in the presence of DCF or/and PAA. The presented adsorption/desorption/aggregation results proved that starting material — fly ash, and Na-P1(C) composite can be successfully applied in wastewater treatment technology. The use of toxic product of coal combustion is very important from the point of view of its management and reduction of its storage problem.

## Data Availability

Not applicable.

## References

[CR1] Akarsubasi AT, Ince O, Kirdar B, Oz NA, Orhon D, Curtis TP, Head IM, Ince BK (2005). Effect of wastewater composition on archaeal population diversity. Water Res.

[CR2] Al-Khateeb LA, Hakami W, Salam MA (2017). Removal of non-steroidal anti-inflammatory drugs from water using high surface area nanographene: Kinetic and thermodynamic studies. J Mol Liq.

[CR3] Azari A, Salari M, Dehghani MH, Alimohammadi M, Ghaffari HR, Sharafi K, Shariatifar N, Baziar M (2017). Efficiency of magnitized graphene oxide nanoparticles in removal of 2,4-dichlorophenol from aqueous solution. J Maz Univ Med Sci.

[CR4] Bandura L, Panek R, Madej J, Franus W (2021). Synthesis of zeolite-carbon composites using high-carbon fly ash and their adsorption abilities towards petroleum substances. Fuel.

[CR5] Badi MY, Esrafili A, Pasalari H, Kalantary RR, Ahmadi E, Gholami M, Azari A (2019). Degradation of dimethyl phthalate using persulfate activated by UV and ferrous ions: optimizing operational parameters mechanism and pathway. J Environ Health Sci Eng.

[CR6] Berizi Z, Hashemi SY, Had M, Azari A, Mahvi AH (2016). The study of non-linear kinetics and adsorption isotherm models for Acid Red 18 from aqueous solutions by magnetite nanoparticles and magnetite nanoparticles modified by sodium alginate. Water Sci Technol.

[CR7] Bhadra BN, Ahmed I, Kim S, Jhung SH (2017). Adsorptive removal of ibuprofen and diclofenac from water using metalorganic framework-derived porous carbon. Chem Eng J.

[CR8] Camargo-Perea AL, Rubio-Clemente A, Peñuela GA (2020). Use of ultrasound as an advanced oxidation process for the degradation of emerging pollutants in water. Water.

[CR9] Carvalho PN, Pirra A, Basto MCP, Almeida CMR (2013). Activated sludge systems removal efficiency of veterinary pharmaceuticals from slaughterhouse wastewater. Environ Sci Pollut Res.

[CR10] Chen G, Ekama GA, van Loosdrecht MCM, Brdjanovic D (2020) Biological wastewater treatment: principles, modelling and design. Chapter 1. IWA Publishing, ISBN electronic: 9781789060362. 10.2166/9781789060362

[CR11] Chibowski S, Wiśniewska M, Marczewski AW, Pikus S (2003). Application of the SAXS and viscosimetry for determination of the thickness of adsorbed polymer layers at the ZrO_2_-polymer solution interface. J Colloid Interf Sci.

[CR12] Correa-Navarro YM, Giraldo L, Moreno-Pirajan JC (2020). Biochar from figue bagasse for removal of caffeine and diclofenac from aqueous solution. Molecules.

[CR13] Correia VM, Stephenson T, Judd SJ (1994). Characterisation of textile wastewaters - a review. Environ Tech.

[CR14] De Luna MDG, Murniati Budianta W, Rivera KKP, Arazo RO (2017). Removal of sodium diclofenac from aqueous solution by adsorbents derived from cocoa pod husks. J Environ Chem Eng.

[CR15] Fijałkowska G, Wiśniewska M, Szewczuk-Karpisz K (2020). Adsorption and electrokinetic studies in kaolinite/anionic polyacrylamide/chromate ions system. Colloids Surf.

[CR16] Fijałkowska G, Szewczuk-Karpisz K, Wiśniewska M (2020b) Anionic polyacrylamide as a substance strengthening the Pb(II) immobilization on the kaolinite surface. Int J Environ Sci Technol 17(2):1101–1112. 10.1007/s13762-019-02546-6

[CR17] Fu F, Wang Q (2011). Removal of heavy metal ions from wastewaters: a review. J Environ Manag.

[CR18] Gebhardt JE, Furstenau DW (1983). Adsorption of polyacrylic acid at oxide/water interfaces. Colloids Surf A.

[CR19] Gogate PR, Pandit AB (2004). A review of imperative technologies for wastewater treatment II: hybrid methods. Adv Environ Res.

[CR20] Hu X, Cheng Z, Sun Z, Zhu H (2017). Adsorption of diclofenac and triclosan in aqueous solution by purified multi-walled carbon nanotubes. Polish J Environ Stud.

[CR21] Huang M, Li Y, Gu G (2010). Chemical composition of organic matters in domestic wastewater. Desalinat.

[CR22] Janusz W (1994). Electrical double layer in the system TiO_2_ (anathase)/aqueous solution of NaCl. Polish J Chem.

[CR23] Jodeh S, Abdelwahab F, Jaradat N, Warad I, Jodeh W (2016). Adsorption of diclofenac from aqueous solution using Cyclamen persicum tubers based activated carbon (CTAC). J Assoc Arab Univ Basic Appl Sci.

[CR24] Khaleque A, Alam MM, Hoque M, Mondal S, Haider JB, Xu B, Johir MAH, Karmakar AK, Zhou JL, Ahmed MB, Moni MA (2020). Zeolite synthesis from low-cost materials and environmental applications: a review. Env Adv.

[CR25] Kümmerer K (2001). Drugs in the environment: emission of drugs, diagnostic aids and disinfectants into wastewater by hospitals in relation to other sources – a review. Chemosphere.

[CR26] Lach J, Szymonik A (2020). Adsorption of diclofenac sodium from aqueous solutions on commercial activated carbons. Des Water Treat.

[CR27] Li X, Ji M, Nghiem LD, Zhao Y, Liu D, Yang Y, Wang Q, Trinh QT, Vo DVN, Pham VQ, Tran NH (2020). A novel red mud adsorbent for phosphorus and diclofenac removal from wastewater. J Mol Liq.

[CR28] M’Pandou A, Siffert B (1987). Polyethylene glycol adsorption at the TiO_2_-H_2_O interface: distortion of ionic structure and shear plane position. Colloids Surf A.

[CR29] Mohammadi L, Kamani H, Asghari A, Mohammadpour A, Golaki M, Rahdar A, Kyzas GZ (2022). Removal of amoxicillin from aqueous media by fenton-like sonolysis/H_2_O_2_ process using zero-valent iron nanoparticles. Molecules.

[CR30] Oshima H (1994). A simple expression for Henry’s function for the retardation effect in electrophoresis of spherical colloidal particles. J Colloid Interf Sci.

[CR31] Panek R, Madej J, Bandura L, Słowik G (2021). Recycling of waste solution after hydrothermal conversion of fly ash on a semi-technical scale for zeolite synthesis. Materials.

[CR32] Panek R, Medykowska M, Szewczuk-Karpisz K, Wiśniewska M (2021). Comparison of physicochemical properties of fly ash precursor, Na-P1(C) zeolite–carbon composite and Na-P1 zeolite—adsorption affinity to divalent Pb and Zn cations. Materials.

[CR33] Panek R, Medykowska M, Wiśniewska M, Szewczuk-Karpisz K, Jędruchniewicz K, Franus M (2021). Simultaneous removal of Pb^2+^ and Zn^2+^ heavy metals using fly ash Na-X zeolite and its carbon Na-X(C) composite. Materials.

[CR34] Petrie B, McAdam EJ, Scrimshaw MD, Lester JN, Cartmell E (2013). Fate of drugs during wastewater treatment. TrAC Trends Anal Chem.

[CR35] Rigueto CVT, Rosseto M, Nazari MT, Ostwald BEP, Alessandretti I, Manera C, Piccin JS, Dettmer A (2021). Adsorption of diclofenac sodium by composite beads prepared from tannery wastes-derived gelatin and carbon nanotubes. J Environ Chem Eng.

[CR36] Sangeetha C, Baskar P (2016). Zeolite and its potential uses in agriculture: a critical review. Agric Rev.

[CR37] Shamsudin MS, Azha SF, Sellaoui L, Badawi M, Bonilla-Petriciolet A, Ismail S (2022). Performance and interactions of diclofenac adsorption using Alginate/Carbon-based Films: experimental investigation and statistical physics modelling. Chem Eng J.

[CR38] Shoppert AA, Longinova IV, Chaikin LI, Rogozhnikov DA (2017) Alkali fusion-leaching method for comprehensive processing of fly ash. KnE Materials Science/Technogen 2017:89–96

[CR39] Skwarek E, Janusz W, Sternik D (2014). Adsorption of citrate ions on hydroxyapatite synthetized by various methods. J Radioanal Nucl Chem.

[CR40] Sotelo JL, Ovejero G, Rodriguez A, Alvarez S, Galan J, Garcia J (2014). Competitive adsorption studies of caffeine and diclofenac aqueous solutions by activated carbon. Chem Eng J.

[CR41] Sugano Y, Sahara R, Murakami T, Narushima T, Iguchi Y, Ouchi C (2005). Hydrothermal synthesis of zeolite A using blast furnance slag. Iron Steel Inst Jpn Int.

[CR42] Szewczuk-Karpisz K, Bogatyrov VM, Galaburda MV, Sokołowska Z (2020). Study on adsorption and aggregation in the mixed system of polyacrylamide, cu(II) ions and innovative carbon–silica composite. Polymers.

[CR43] Szewczuk-Karpisz K, Tomczyk A, Celińska M, Sokołowska Z, Kuśmierz M (2021). Carboxin and diuron adsorption mechanism on sunflower husks biochar and goethite in the single/mixed pesticide solutions. Materials.

[CR44] Szewczuk-Karpisz K, Wiśniewska M, Myśliwiec D (2015). Albumin adsorption influence on the stability of the mesoporous zirconia suspension. J Ind Eng Chem.

[CR45] Tomul F, Arslan Y, Başoğlu FT, Babuçcuoğlu Y, Tran HN (2019). Efficient removal of anti-inflammatory from solution by Fe-containing activated carbon: adsorption kinetics, isotherms, and thermodynamics. J Environ Manag.

[CR46] Van Tran T, Nguyen DTC, Le HTN, Vo DVN, Nanda S, Nguyen TD (2020). Optimization, equilibrium, adsorption behavior and role of surface functional groups on graphene oxide-based nanocomposite towards diclofenac drug. J Environ Sci.

[CR47] Viotti PV, Moreira WM, Andreo dos Santos OA, Bergamasco R, Salcedo Vieira AM, Vieira MF (2019). Diclofenac removal from water by adsorption on Moringa oleifera pods and activated carbon: mechanism, kinetic and equilibrium study. J Clean Prod.

[CR48] Wajima T, Yoshizuka K, Hirai T, Ikegami Y (2008). Synthesis of zeolite X from waste sandstone cake using alkali fusion method. Mater Trans.

[CR49] Wiśniewska M, Nosal-Wiercińska A, Dąbrowska I, Szewczuk-Karpisz K (2013). Effect of the solid pore size on the structure of polymer film at the metal oxide/polyacrylic acid solution interface – Temperature impact. Micropor Mesopor Mater.

[CR50] Wiśniewska M, Chibowski S, Urban T (2015). Impact of polyacrylamide with different contents of carboxyl groups on the chromium (III) oxide adsorption properties in aqueous solution. J Hazard Mat.

[CR51] Wiśniewska M, Chibowski S, Urban T (2016). Adsorption properties of the nanozirconia/anionic polyacrylamide system -effects of surfactant presence, solution pH and polymer carboxyl groups content. Appl Surf Sci.

[CR52] Wiśniewska M, Fijałkowska G, Szewczuk-Karpisz K (2018). The mechanism of anionic polyacrylamide adsorption on the montmorillonite surface in the presence of Cr(VI) ions. Chemosphere.

[CR53] Wiśniewska M, Fijałkowska G, Nosal-Wiercińska A, Franus M, Panek R (2019). Adsorption mechanism of poly(vinyl alcohol) on the surfaces of synthetic zeolites: sodalite, Na-P1 and Na-A. Adsorption.

[CR54] Wiśniewska M, Chibowski S, Urban T, Terpiłowski K (2019). Investigations of chromium(III) oxide removal from the aqueous suspension using the mixed flocculant composed of anionic and cationic polyacrylamides. J Hazard Mat.

[CR55] Wiśniewska M, Nowicki P (2020). Peat-based activated carbons as adsorbents for simultaneous separation of organic molecules from mixed solution of poly(acrylic acid) polymer and sodium dodecyl sulfate surfactant. Colloids Surf A.

[CR56] Yamashita N, Yasojima M, Nakada N, Miyajima K, Komori K, Suzuki Y, Tanaka H (2006). Effects of antibacterial agents, levofloxacin and clarithromycin, on aquatic organisms. Water Sci Tech.

[CR57] Yousefzadeh S, Ahmadi E, Gholami M, Ghaffari HR, Azari A, Ansari M, Miri M, Sharafi K, Rezaei S (2017). A comparative study of anaerobic fixed film baffled reactor and up-flow anaerobic fixed film fixed bed reactor for biological removal of diethyl phthalate from wastewater: a performance, kinetic, biogas, and metabolic pathway study. Biotechnol Biofuels.

[CR58] Zhang Y, Geißen SU, Gal C (2008). Carbamazepine and diclofenac: removal in wastewater treatment plants and occurrence in water bodies. Chemosphere.

[CR59] Zuccato E, Castiglioni S, Fanelli R (2005). Identification of the pharmaceuticals for human use contaminating the Italian aquatic environment. J Hazard Mat.

